# Tyr-Tyr-Glu Tripeptide for Regeneration of Articular Cartilage through Chondrogenic Differentiation of Mesenchymal Stem Cells

**DOI:** 10.34133/bmr.0272

**Published:** 2025-11-14

**Authors:** Ye Lin Kim, Soyoun Um, Madhumita Patel, Yeon-Ju Jung, Sun-Shin Cha, Jihee Kim, Soo Young Lee, Byeongmoon Jeong

**Affiliations:** ^1^ Department of Chemistry and Nanoscience, Gradutate Program in Innovative Biomaterials Convergence, Ewha Womans University, Seoul 03760, Korea.; ^2^ R&D Division, TODD Phaarm Co. Ltd., Seoul 03760, Korea.; ^3^ Department of Life Sciences and Multitasking Macrophage Research Center, Ewha Womans University, Seoul 03760, Korea.

## Abstract

Cell-based therapy accompanying articular cartilage regeneration is very promising as one of osteoarthritis (OA) treatments. Here, we report that Tyr-Tyr-Glu (YYE) is effective for articular cartilage regeneration through chondrogenic differentation of mesenchymal stem cells (MSCs). The expression of COL II, COMP, ACAN, and COL X at the mRNA and/or protein levels, together with Alcian Blue and Safranin O staining of 3-dimensionally cultured cell pellets, consistently demonstrated that YYE is an effective chondrogenic promoter of MSCs. Western blot, immunofluorescence, and molecular docking studies suggested that the chondrogenic up-regulation by YYE is mediated through YYE binding to FC-1, which induces translocation of core-binding factor β subunit (CBFβ) from the cytosol into the nucleus, followed by up-regulation of runt-related transcription factor 1 (RUNX1), which is a key factor for chondrogenic differentiation of MSCs. In particular, intra-articular injection of YYE to mouse OA models led to a significant improvements of OA as indicated by lowering OARSI grades and subchondral bone plate thickness, and thus proved its articular cartilage regeneration efficacy.

## Introduction

Articular cartilage consists of chondrocytes, proteoglycans, proteins, and water. It is an avascular/aneural tissue and does not regenerate when the damage is above a critical size [1]. In particular, osteoarthritis (OA) caused by degeneration of articular cartilage is observed in more than 29% of adults who are 60 years old or more, and thus, OA will be a more serious disease due to the increase of the older population [2,3]. As of 2020, OA affects 650 million people worldwide for aged 40 and older [4]. The Advanced Research Projects Agency for Health (ARPA-H) of the United States initiated the Novel Innovations for Tissue Regeneration in Osteoarthritis (NITRO) Program in 2023 to challenge OA as the first target disease [5]. Current treatments of OA are mostly based on the pain-relief therapy; however, fundamental treatments based on regeneration of articular cartilage are rare. Invossa is a cell-mediated gene therapy product consisting in a ratio of 3:1 of human allogenic chondrocytes and retrovially transduced chondrocytes to overexpress transforming growth factor-β1 (TGF-β1). Invossa had been approved in Korea in 2015 as an intra-articular (IA) injection for OA patients, but was withdrawn in 2019 due to the mislabeled cell types and is under phase III clinical trial in the United States [6,7]. Cartistem of Medipost was clinically approved in 2011 by Food and Drug Administration of Korea (KFDA), which uses human umbilical cord blood-derived mesenchymal stem cells (hUCB-MSCs) for OA patients. However, paracrine effects of MSC secretomes are responsible for improvement of OA through pain relief while accompanying minor cartilage tissue regeneration [8,9]. The efficient differentiation of stem cells into chondrocytes is still a concern in most OA treatments including Cartistem. To enhance chondrogenic differentiation of MSCs, various approaches have been attempted. Among them, the approach using well-defined factors to trigger chondrogenic differentiation of stem cells is considered to be the most promising method [10]. Many factors that promote chondrogenic differentiation of MSCs have been developed, including TGF-β1, fibroblast growth factor-2 (FGF-2), epidermal growth factor, insulin-like growth factor-1, bone morphogenic protein-2,4,7, platelet-derived growth factor, and interleukin-1β [10–25]. Compared with these protein- or polypeptide-based growth factors, small molecules are chemically well-defined and offer greater reproducibility both in production and in their in vitro and in vivo efficacy as promoters of MSC chondrogenic differentiation. Notably, TGF-β1, one of the most effective proteins for promoting MSC chondrogenesis, has been reported to induce synovial fibrosis, osteophyte formation, and endochondral ossification [16–18]. FGF-2 similarly can cause histopathological clonal cluster formation of chondrocytes, inhibit proteoglycan synthesis, and elevate inflammatory markers such as matrix metalloproteinases, thereby accelerating OA development [19]. Since the report of kartogenin (KGN), an oxopiperazine derivative of 5{i,2}, and pralatrexate, many studies have sought to discover effective small-molecule chondrogenic promoters [20–23]. KGN completed a phase I clinical trial in the United States in 2021 for cartilage regeneration [24].

FC-1 is the C-terminal fragment of filamin A that interacts with the core-binding factor β subunit (CBFβ). Binding of a compound to FC-1 is known to promote chondrogenic differentiation of MSCs [20,23]. The binding site of FC-1 needs both aromatic ring and carboxylate for π–π interactions and hydrogen bonding as will be discussed. In addition, strong chondrogenic differentiation promoter compounds of KGN, an oxopiperazine derivative of 5{i,2}, and pralatrexate have both aromatic ring and carboxylic acid in their structures [20,21,23].

In this study, a series of tripeptides with aromatic group (F, Y, W) and carboxylic acid (D, E) were selected to target the FC-1 binding mechanism for chondrogenic differentiation of MSCs. To evaluate the chondrogenic effectiveness of the tripeptides at a level comparable to KGN (positive control) reported in *Science*, all experiments were conducted using tripeptide solutions at 10 μM, the concentration of which was shown to be effective in the literature [20]. In the first round, the top 3 tripeptides exhibiting enhanced biomarker expression for chondrogenic differentiation of MSCs were selected. In the second round, the selected 3 tripeptides were investigated in detail by 3-dimensional (3D) pellet culture over 21 d and a final drug candidate was selected. In addition, the effectiveness of the final drug candidate for articular cartilage regeneration was investigated in an OA mouse model.

## Materials and Methods

### Chemicals

All peptides used in the experiments were purchased from Koma Biotech Inc., Korea. The purity of the peptides was >98% [high-performance liquid chromatography (HPLC) traces were provided in Fig. S1]. KGN, which was used as a positive control compound to induce chondrogenic differentiation of MSCs, was purchased from Sigma-Aldrich, USA. Fetal bovine serum (FBS), penicillin, and streptomycin were purchased from Hyclone, USA. Ascorbate-2-phosphate, l-proline, and insulin–transferrin–selenium (ITS) Premix containing bovine insulin, transferrin, sodium selenite, linoleic acid, dexamethasone, and bovine serum albumin (BSA) were purchased from Sigma-Aldrich, USA.

### 2D cell culture

Tonsil-derived mesenchymal stem cells (TMSCs) were donated by the Ewha Womans University Mokdong Hospital (Seoul, Korea). They were isolated from a 6-year-old male donor after tonsillectomy in accordance with the institutional review board guideline. The stem cells were 2D cultured on plates at 37 °C until passage 5 in a growth medium of high-glucose Dulbecco’s modified Eagle’s medium (DMEM) (Hyclone, USA) supplemented with 10.0% (v/v) FBS, 1.0% (v/v) antibiotic/antimitotic solution (Gibco, USA), and 1.0% (v/v) penicillin/streptomycin solution under 5% CO_2_ conditions. The stem cells (passage 6) were subsequently 2D cultured at 37 °C under 5% CO_2_ conditions. After 24 h, the growth medium (3.0 ml) supplemented with 10.0% (v/v) FBS, 1.0% (v/v) antibiotic/antimitotic solution (Gibco, USA), and 1.0% (v/v) penicillin/streptomycin was replaced by the same growth medium except that FBS, called starvation medium, and individual tripeptide or KGN was added to the solution. Control (negative) experiments were performed in the absence of individual tripeptides or KGN. A KGN-containing system is used in the positive control experiment. The medium was replaced every other day for 7 d. The same numbers of the stem cells (2.5 × 10^5^ cells per well) were used for each system, and each experiment was performed in triplicate (*N* = 3).

#### Toxicity of tripeptides

A cell counting kit-8 (CCK-8) solution (10.0%, Dojindo, Japan) was prepared in high-glucose DMEM (Hyclone, USA) supplemented with 1.0% (v/v) penicillin/streptomycin solution. The solution (0.5 ml) replaced the media of each cell culture well in which the stem cells had been incubated for 3 d by using growth medium supplemented with individual tripeptide (10 μM) or KGN, 1.0% (v/v) antibiotic/antimitotic solution (Gibco, USA), and 1.0% (v/v) penicillin/streptomycin solution. For CCK-8 assay, the medium is replaced by CCK-8 solution and the cells were incubated for 3 h at 37 °C under the 5% CO_2_ conditions. Then, absorbance of the sample was measured at 450 nm relative to 655 nm by using a microplate reader (iMark, Bio-Rad, USA). Absorbance values were converted to cell viability relative to the control group, defined as cultures without tripeptides or KGN. Cell viability after 3 d of incubation was expressed relative to that at day 0, which was set as 100%. For Live/Dead assay, the media were replaced by phosphate-buffered saline (PBS) containing ethidium homodimer-1 (4.0 μM) and calcein acetoxy methyl ester (2.0 μM), and the cells were incubated for 15 min. The viability of the stem cells was investigated with the Live/Dead kit (Molecular Probes, Life Technologies, USA) by using an Olympus IX71 fluorescence microscope and the Olympus DP2-BSW software (Olympus Corporation, Tokyo, Japan).

#### Screening of tripeptides

The stem cells were 2D cultured in high-glucose DMEM (Hyclone, USA) supplemented with 1.0% (v/v) penicillin/streptomycin solution and tripeptide (10 μM) or KGN [20]. The stem cells were incubated for 7 d. The chondrogenic biomarker expression was investigated at the mRNA and/or protein levels.

### 3D cell pellet culture

The stem cells (passage 6) were enzymatically detached via trypsin treatments and counted by a hemocytometer. The tube was centrifuged at 500*g* relative centrifugal force (RCF) for 10 min and incubated at 37 °C under 5% CO_2_ conditions for 24 h. A cell pellet consisting of 3.0 × 10^5^ cells was formed in a conical polypropylene tube (15 ml). The pellets were cultured at 37 °C in a high-glucose DMEM supplemented with 1.0% (v/v) antibiotic/antimitotic solution, 1.0% (v/v) penicillin/streptomycin (medium A), medium A containing additional individual tripeptides at 10 μM (medium B), or medium A containing additional KGN at 10 μM (medium C) for 3 d under 5% CO_2_ conditions. Then, the medium was replaced by a chondrogenic induction medium containing a high-glucose DMEM supplemented with 1.0% (v/v) antibiotic/antimitotic solution, 1.0% (v/v) penicillin/streptomycin, 50 μg/ml ascorbate-2-phosphate, 40 μg/ml l-proline, 100 nM dexamethasone, and 1% ITS + Premix with a final concentration of 10 μg/ml bovine insulin, 5.5 μg/ml transferrin, 5 μg/ml sodium selenite, 4.7 μg/ml linoleic acid, and 0.5 mg/ml BSA. The cells were incubated over 21 d. The chondrogenic induction medium was replaced every 3 d. The experiments were performed in triplicate for each system. The cell pellets were fixed in formalin aqueous solution (10%, v/v) and embedded in paraffin. Subsequently, these pellets were sectioned to a thickness of 7 μm using a microtome. Then, sliced samples were then deparaffinized with xylene and evaluated via Alcian Blue, Safranin O, and antibodies staining for visualization of sulfated glycosaminoglycan (sGAG), proteoglycan, and type II collagen (COL II), respectively.

#### Alcian Blue staining

Pellets as well as sliced pellets were treated with Alcian Blue solution [1.0% w/v in acetic acid aqueous solution (3.0% v/v), Sigma-Aldrich, USA] and Nuclear Fast Red aqueous solution (0.1% w/v, Sigma-Aldrich, USA).

#### Safranin O staining

Sliced pellet samples were treated with a Fast Green FCF aqueous solution (0.001% w/v, Sigma-Aldrich, USA) and Safranin O aqueous solution (0.1% w/v Acros Organics, USA) in accordance with the previous protocol [26].

#### Immunofluorescence

Sliced pellet samples were permeabilized by Triton (0.1% v/v in PBS) and treated with BSA (1.0% w/v in PBS, Sigma, USA). COL II was investigated using goat anti-mouse immunoglobulin G (IgG) H&L Alexa Fluor 594 (Abcam, USA) and goat anti-rabbit IgG H&L Alexa Fluor 594 (Abcam, USA). The nucleus and actin were also stained with 4′,6-diamidino-2-phenylindole (DAPI) (Molecular Probes, USA) and phalloidin (Abcam, USA), respectively.

### RNA extraction and real-time RT-PCR

Total RNA was extracted from the cell by using a TRIZOL reagent (Invitrogen, USA). The mRNA concentration was assayed by a NanoDrop 2000 spectrophotometer (Thermo Scientific, USA). cDNA was synthesized by a ReverTra Ace qPCR RT kit (Toyobo, Japan). The mRNA expression levels were assayed through real-time reverse transcription polymerase chain reaction (RT-PCR) (CFX96, Bio-Rad, USA) using iQ SYBR Green Supermix (Bio-Rad, USA). The expression level of genes was calculated as 2^−△△Ct^, where ΔΔCt = (Gene A − GAPDH) − (Gene A – GAPDH)_Control_. The primer sequences were listed in Table S1.

### Mechanism study

Chondrogenic promotion by tripeptides was investigated by in silico modeling and expression of target proteins [CBFβ and runt-related transcription factor 1 (RUNX1)] in the cytosol and nucleus.

#### Molecular docking study between the tripeptide and FC-1 fragment of filamin A

To make the in silico models of FC-1 complexed with YYE, WFE, WLE, and KGN, we generated the model structure of the FC-1 fragment by using the Swiss-Model server, and the structures of ligands (YYE, WFE, WLE, and KGN) were prepared using the ChemDraw 3D software (Perkin Elmer informatics, MA, USA) [27,28]. Molecular docking of ligands against FC-1 was performed using AutoDock Vina version 1.5.6 rc2, an open-source software developed at The Scripps Research Institute, USA [29].

#### Western blotting CBFβ

TMSCs were treated with tripeptide (10 μM) in serum-free growth medium after overnight starvation. KGN was also used as a control. After 12 h of incubation, cytosolic and nuclear proteins from the stem cells were collected with a nuclear/cytosolic fraction kit (Cell Biolabs Inc., USA), and total proteins were collected in cell extraction buffer (Invitrogen, USA) with protease inhibitors and phenyl methane sulfonyl fluoride. Cytosolic, nuclear, and total proteins were detected with anti-CBFβ (Cell Signaling Technology 62184, 1:1,000, USA) primary antibody, followed by incubation in anti-rabbit IgG horseradish peroxidase (HRP)-linked secondary antibody (Cell Signaling Technology, 7074, 1:1,000, USA) for CBFβ. The proteins were detected with an enhanced chemiluminescent substrate (Bio-Rad, USA) using Amersham Imager 600 (GE, UK) and quantified by ImageJ software (National Institutes of Health, USA).

#### Immunofluorescence study of CBFβ and RUNX1

The stem cells were treated with tripeptides (10 μM) in serum-free growth medium for 12 h followed by overnight starvation. KGN was also used as a control. Thereafter, protein expression of CBFβ and RUNX1 was evaluated by the immunofluorescence assay kits. Cells were fixed and permeabilized with paraformaldehyde and Triton, respectively. Subsequently, cells were blocked with 1.0% BSA and incubated with anti-CBFβ primary antibody (Abcam, ab33516, 1:400, USA) and RUNX1 primary antibody (Santa Cruz Biotechnology, sc365644, 1:200, USA). The cells were then incubated in anti-rabbit IgG H&L secondary antibodies (Abcam, ab150084, 1:200, USA) and anti-mouse IgG H&L secondary antibodies (Abcam, ab150116, 1:200, USA) for CBFβ and RUNX1, respectively. The nuclei were stained with DAPI (Molecular Probes, USA). Fluorescent images were captured on an Olympus IX71 fluorescence microscope (Olympus Corporation, Tokyo, Japan) and quantified by ImageJ software (National Institutes of Health, USA).

### Animal study

#### DMM surgery-induced murine OA and treatment with YYE

Male C57BL/6J mice, aged between 10 and 11 weeks, were utilized as the subjects in this study. The mice were obtained from DBL, South Korea, and were maintained within a controlled environment. They were housed in pathogen-free barrier facilities, with each cage accommodating 5 or fewer mice. The housing conditions were set at a temperature range of 24 to 26 °C, humidity levels spanning 30% to 60%, and a light/dark cycle of 12 h each. The assignment of mice to distinct experimental groups was carried out in a randomized manner. OA was induced in C57BL/6J wild-type mice through the implementation of destabilization of medial meniscus (DMM) surgery, as previously outlined [30]. This technique involved the surgical excision of the medial meniscus ligament from the knee joint of right hind limb. For comparative purposes, sham-operated mice underwent a similar surgical procedure on the opposite knee, although without the removal of the medial meniscus ligament. The designated sacrifice point for the mice was 9 weeks post-surgery. In order to explore potential interventions, the mice received IA injections. These injections (10 μl) encompassed 0.1 or 10 μM concentrations of KGN, YYE, or a control vehicle (Veh, PBS). Three mice were used for each group. The injection regimen consisted of once-per-week administration, spanning a total of 4 injections. This treatment initiation occurred 5 weeks after the surgical procedure, effectively targeting the latter half of the OA period. The final IA injection took place 1 week prior to the designated sacrifice at the 8-week mark.

#### Histological analysis of OA

The knee articulations of mice were immobilized in a 10% formaldehyde solution at temperatures of 4 °C for a duration surpassing 24 h. Subsequently, they underwent decalcification within a 0.5 M ethylenediamine tetraacetic acid (EDTA) solution in PBS (pH 7.4) over the course of a 2-week period. The processed samples were then embedded within paraffin, following which they were sectioned into slices measuring 5 μm in thickness. The tissue sections were subjected to staining procedures utilizing hematoxylin and eosin, as well as 0.1% Safranin O (s8884; Sigma-Aldrich, St Louis, MO, USA) and 0.05% Fast Green FCF (f7258; Sigma-Aldrich, St Louis, MO, USA). The presence of sclerosis and degradation within the articular cartilage was discerned through the utilization of Safranin O staining. Quantification of these features was carried out employing OsteoMeasureXP (OsteoMetrics Inc., Atlanta, GA, USA), Image-Pro Plus (v4.5, Media Cybernetics Inc., Rockville, USA), Adobe Photoshop (v9.0, San Jose, CA, USA), and an Olympus DP72 charge-coupled device camera (v2.1, Olympus Corporation, Tokyo, Japan). To ascertain the extent of articular cartilage damage, a standardized OA grading system known as Osteoarthritis Research Society International (OARSI) grades (ranging from 0 to 6) was employed [31]. The maturity of osteophytes and the presence of synovitis were also evaluated [32]. Additionally, measurements of subchondral bone sclerosis involved the assessment of subchondral bone plate (SBP) thickness. The quantified data were presented as the mean ± standard error of the mean.

### Statistical analysis

Statistical significance was analyzed by one-way analysis of variance (ANOVA) with Tukey tests. * and ** indicate *P* < 0.05 and *P* < 0.01, respectively.

## Results and Discussion

### Preliminary screening of tripeptides

**Fig. 1.**
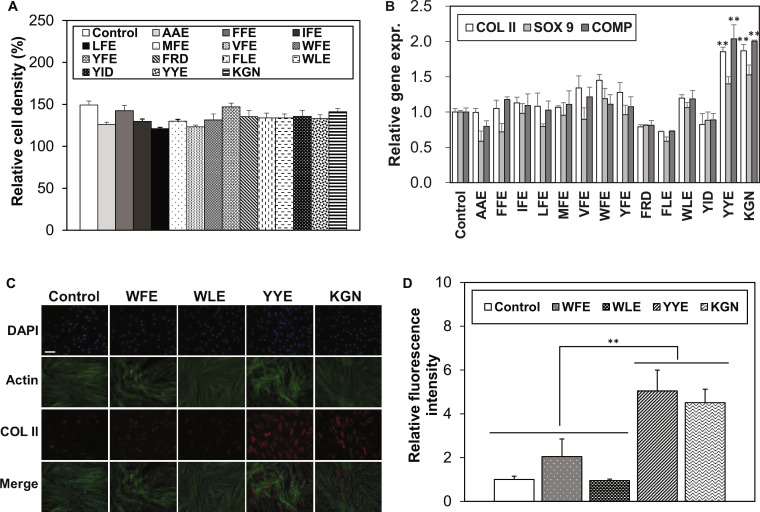
Screening of tripeptides as a promoter compound for chondrogenic differentiation of the MSCs. *N* = 3. The control was defined as a cell culture system in DMEM without promoter compounds. (A) Toxicity test of tripeptides at 10 μM. Change in the cell density of the MSCs was analyzed by the CCK-8 assay. The cell density at day 0 was set to 100% for comparison with cultures containing tripeptides after 3 d of incubation. (B) Chondrogenic biomarker expression at the mRNA level after 7 d of incubation in DMEM containing the tripeptides (10 μM). mRNA expression level was assayed through real-time RT-PCR. (C) Immunofluorescence image of COL II protein. Scale bar, 50 μm. (D) Semiquantitative analysis of the fluorescence intensity in (C). ** in (B) and (D) indicates *P* < 0.01 compared with control.

AAE, FFE, IFE, LFE, MFE, VFE, WFE, YFE, FRD, FLE, WLE, YID, and YYE were selected in the first round of screening. HPLC traces of the tripeptides indicate their purities to be >98% (Fig. S1). For preliminary toxicity test of the compounds, MSCs were 2D cultured in a serum-free Dulbecco’s modified eagle medium (DMEM) in the presence (10 μM) or absence of the above tripeptides over 3 d [20,21,23]. Live/Dead staining confirmed that all cells remained viable (Fig. S2), and the cell density measured by CCK-8 increased 1.3- to 1.5-fold over 3 d of incubation. However, this increase was not significantly different among the groups containing tripeptides (Fig. 1A). These data indicate that the above tripeptides are not cytotoxic in the experimental concentration at 10 μM. Targeting an improved effectiveness of the tripeptides compared with KGN (positive control), chondrogenic biomarker expression was studied at the mRNA level for COL II, SRY-box 9 (SOX 9), and cartilage oligomeric matrix protein (COMP) in tripeptide or KGN culture solutions. These biomarkers were reported to develop significantly over 7 d of culture during chondrogenic differentiation of MSCs [33]. Therefore, 7 d of culture was used for the prescreening purpose of the first round. The chondrogenic biomarkers at the mRNA level were enhanced in the presence of WFE, WLE, and YYE as well as KGN (positive control) (Fig. 1B). In particular, YYE and KGN served as an excellent promoter compound in the chondrogenic biomarker expression. To confirm the chondrogenic biomarker expression at the protein level, immunofluorescence study was performed for the most generally accepted chondrogenic biomarker of COL II protein. The high population of red stained images indicates that the COL II protein expression was significantly enhanced by using YYE as well as KGN (Fig. 1C and D).

**Fig. 2.**
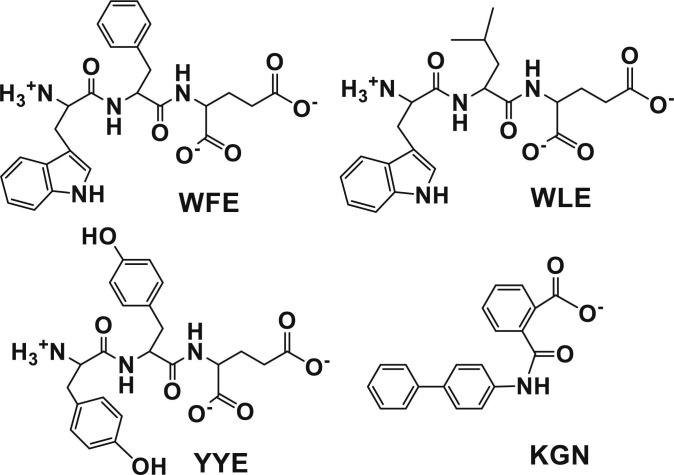
Chemical structures of potential candidate compounds for promoting chondrogenic differentiation of MSCs. The chemical structure of KGN, used as a positive control, is also shown. All chemical structures were drawn using ChemDraw Software (Revvity Signals Inc., USA).

Based on the above prescreening study, WFE, WLE, and YYE were selected as potential candidates as chondrogenic promoter compounds of MSCs. The second round of screening for chondrogenic differentiation of MSCs using the selected tripeptides was studied in detail by using a 3D pellet culture over the extended time period of 21 d. The chemical structures of WFE, WLE, YYE, and KGN (positive control) are shown in Fig. 2.

### 3D pellet culture of MSCs in the presence of tripeptides

**Fig. 3.**
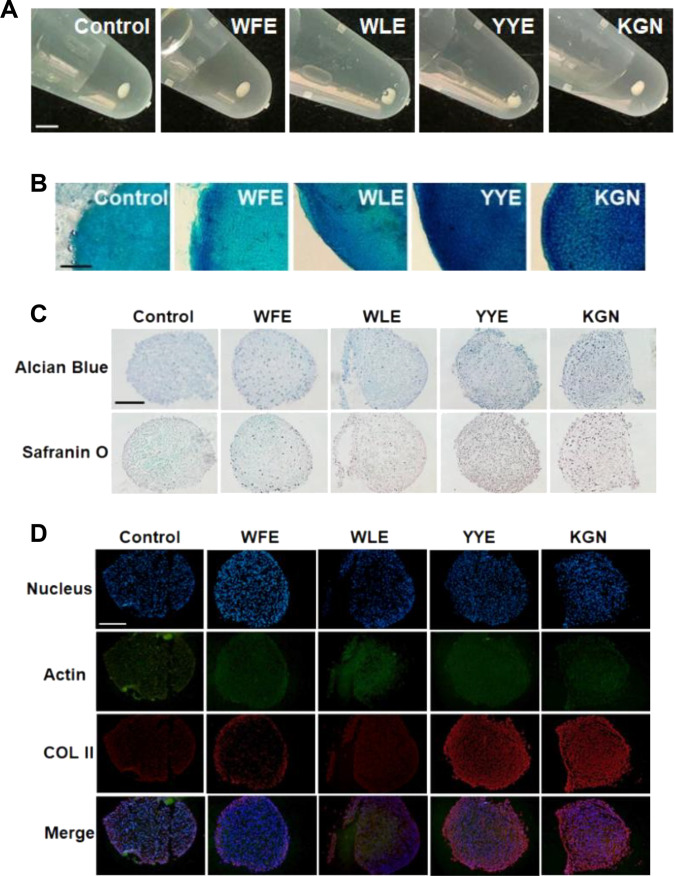
(A) Photos of cell pellets after 21 d of cell culture. Scale bar, 2 mm. (B) Alcian Blue staining of the pellets cultured for 21 d. Scale bar, 0.2 mm. (C) Alcian Blue and Safranin O staining of sliced pellets (thickness ~7 μm). Chondrogenic biomarkers of sGAG (Alcian Blue assay) and proteoglycan (Safranin O assay) were stained in blue and red, respectively. (D) Immunofluorescence images of COL II of the sliced pellets stained. COL II is stained in red. The scale bars in (C) and (D) are 0.5 mm.

Considering in vivo application of the compound in articular cartilage of OA patients, the microenvironments can be simulated with a 3D culture system better than a 2D culture system in chondrogenic induction medium. The cell pellets cultured for 21 d were assayed for the expression of chondrogenic biomarkers of sGAG, proteoglycan, and COL II [34–36]. Figure 3A shows the photos of cell pellets cultured for 21 d. The cell pellets cultured in the presence of YYE or KGN exhibited a deep blue color of sGAG by the Alcian Blue assay (Fig. 3B). The cell pellets were sliced with a thickness of 7 μm to allow facile permeation of the staining agents. The sliced cell pellets were also distinguished by blue (sGAG) and red (proteoglycan) in the Alcian Blue and Safranin O staining, respectively, for the cell pellets cultured in the presence of YYE and KGN (Fig. 3C). The YYE- and KGN-treated systems were distinguished for their red staining by COL II in contrast with the control, WFE-treated, or WLE-treated systems (Fig. 3D).

**Fig. 4.**
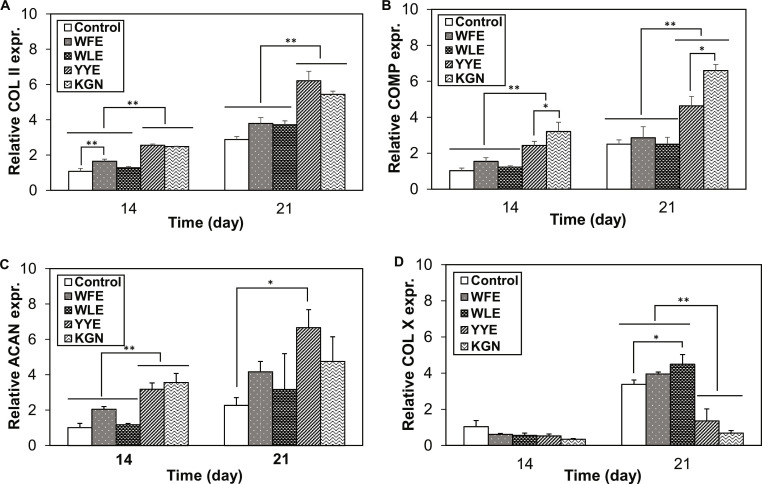
Biomarkers at the mRNA level assayed by real-time RT-PCR. Chondrogenic biomarkers [COL II (A), COMP (B), and ACAN (C)] and hypertropic biomarker of COL X (D), indicating that the chondrogenic biomarker expression is significantly higher in the presence of YYE and KGN than control. *N* = 3 for each group. * and ** in (B) to (D) indicate *P* < 0.05 and *P* < 0.01, respectively, compared with control.

The chondrogenic biomarker expressions for COL II, COMP, and aggrecan (ACAN) were also analyzed for the pellet culture systems at the mRNA level. These chondrogenic mRNAs of COL II, COMP, and ACAN were all highly expressed in the systems treated with YYE and KGN (Fig. 4A to C). Compared with control system that was not treated with tripeptides, YYE 2-fold, 2-fold, and 3-fold increased the COL II, COMP, and ACAN expression at the mRNA levels, respectively, after 21 d of pellet culture, suggesting the effectiveness of YYE in driving chondrogenic differentiation of the MSCs [35,37]. The expressions of chondrogenic biomarkers at the mRNA level treated with YYE were comparable with those of the system treated with KGN, a positive control. COL X is a biomarker related to the hypertrophy of the cells, which represents an intermediate state leading to the osteogenic differentiation [26,38,39]. In this regard, YYE and KGN are specific in enhancing chondrogenic differentiation and suppressing osteogenic differentiation of the MSCs, compared with control, which does not contain tripeptides (Fig. 4D). Compared with the control system, YYE decreased the COL X mRNA expression levels by less than half after 21 d of pellet culture.

### Mechanism of action

**Fig. 5.**
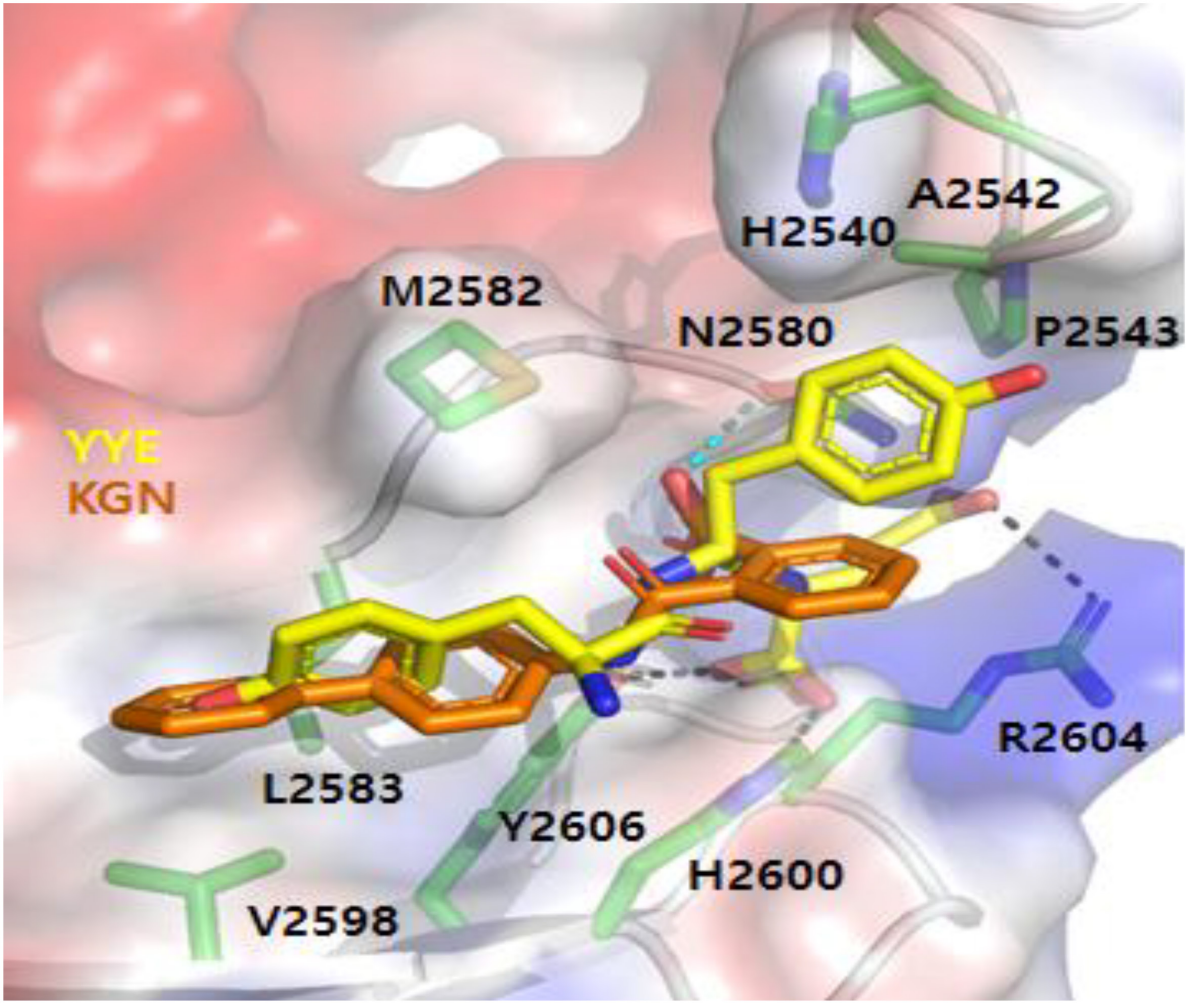
In silico model of YYE (yellow) and KGN (orange) docked to FC-1. Residues of FC-1 interacting with YYE are shown as green sticks on a light gray cartoon and veiled by a transparent surface. Negatively charged, positively charged, and neutral surface areas of FC-1 are colored red, blue, and white, respectively. Black dashed lines indicate electrostatic interactions between YYE and FC-1, while the cyan dashed line represents the interactions between KGN and FC-1. Molecular docking was performed using the AutoDock Vina.

The mechanism of chondrogenic up-regulation by YYE was studied by a molecular docking study. Filamin A interacts with CBFβ [20,23,26]. Binding of a candidate compound to FC-1 of filamin A initiates chondrogenic differentiation of the stem cells by disrupting the interactions between FC-1 and CBFβ. The liberated CBFβ is supposed to enter the nucleus. To get insights into the binding mode of YYE to FC-1, we generated the in silico model of the FC-1/YYE complex. YYE is docked into the space between the edges of 2 β-sheets in FC-1 with −7.0 kcal/mol of the binding free energy (Fig. 5). Phenolic side chains of 2 tyrosine residues in YYE make hydrophobic interactions and π–π interactions, whereas the 2 carboxylate groups of glutamate mediate electrostatic interactions. The pendant carboxylate group of glutamate forms a salt bridge with the guanidinium group of Arg^2604^. The terminal carboxylate group interacts with the His^2600^ imidazole group and the Tyr^2606^ hydroxyl group. To compare the binding mode of YYE to that of KGN, we also built up the in silico FC-1/KGN complex model. As expected, KGN occupies the same space to which YYE binds. The biphenyl group of KGN contacts with hydrophobic interactions and π–π interactions with FC-1, and the carboxylate group forms a hydrogen bond with Asn^2580^. The binding free energy of KGN with FC-1 was calculated to be −6.3 kcal/mol. Considering the location of the binding site on the surface of FC-1, the loss of polar interactions should negatively affect the binding affinity. To compare the binding affinity with other tripeptides, we also built up in silico models of the FC-1/WFE and FC-1/WLE complexes. The binding energies of WFE and WLE were −6.0 and −6.1 kcal/mol, respectively. Higher binding affinity of YYE correlated to the greater expression of chondrogenic differentiation biomarkers.

**Fig. 6.**
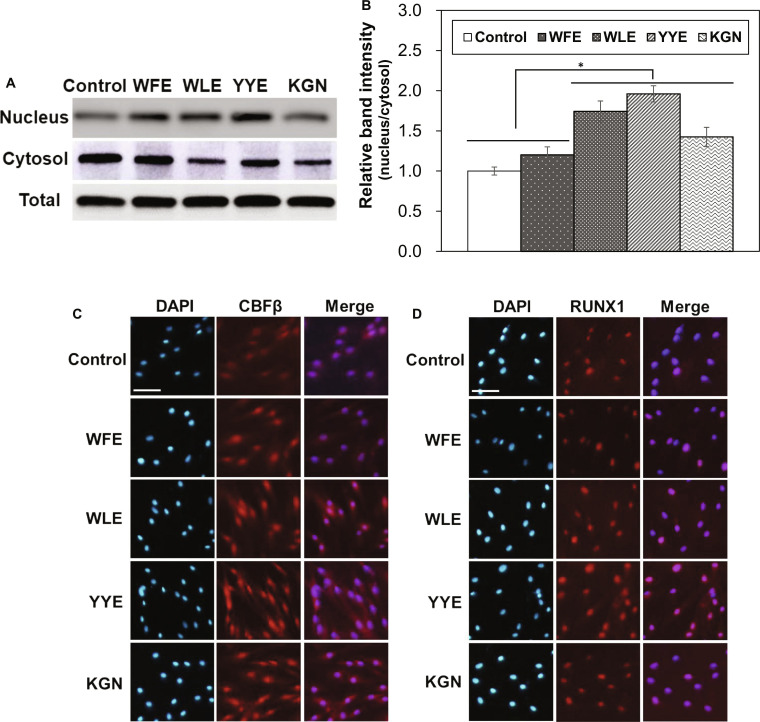
Western blot images of CBFβ (A) and their relative intensity in the nucleus to cytosol (B). *P < 0.05, compared with control. *N* = 3. Immunofluorescence images of CBFβ (C) and RUNX1 (D). Scale bar, 50 μm.

A chondrogenic enhancement mechanism of MSCs was proposed by nuclear translocation of CBFβ, followed by up-regulation of RUNX1 in the cell [20,40,41]. After the treatments of the MSCs by chondrogenic promoter compounds, the expression of nuclear translocation of CBFβ was investigated by the Western blot and immunofluorescence assay. The band intensity of CBFβ at the nucleus relative to that at the cytosol increased by treatments of WLE, YYE, and KGN (Fig. 6A and B). In particular, the relative band intensity of CBFβ in the nucleus treated by YYE 2-fold increased than that of untreated control.

Immunofluorescence images of CBFβ also exhibited high expression of CBFβ in the nucleus of the MSCs treated by YYE (Fig. 6C). RUNX1 expression was assayed by immunofluorescence study of anti-RUNX1. The fluorescence images of RUNX1 demonstrated that fluorescence intensity increased by the treatments of YYE (Fig. 6D). These data suggest that both KGN and YYE have a similar chondrogenic promotion mechanism of MSCs mediated through binding to FC-1, nuclear translocation of CBFβ, and up-regulation of RUNX1.

### In vivo cartilage tissue regeneration

**Fig. 7.**
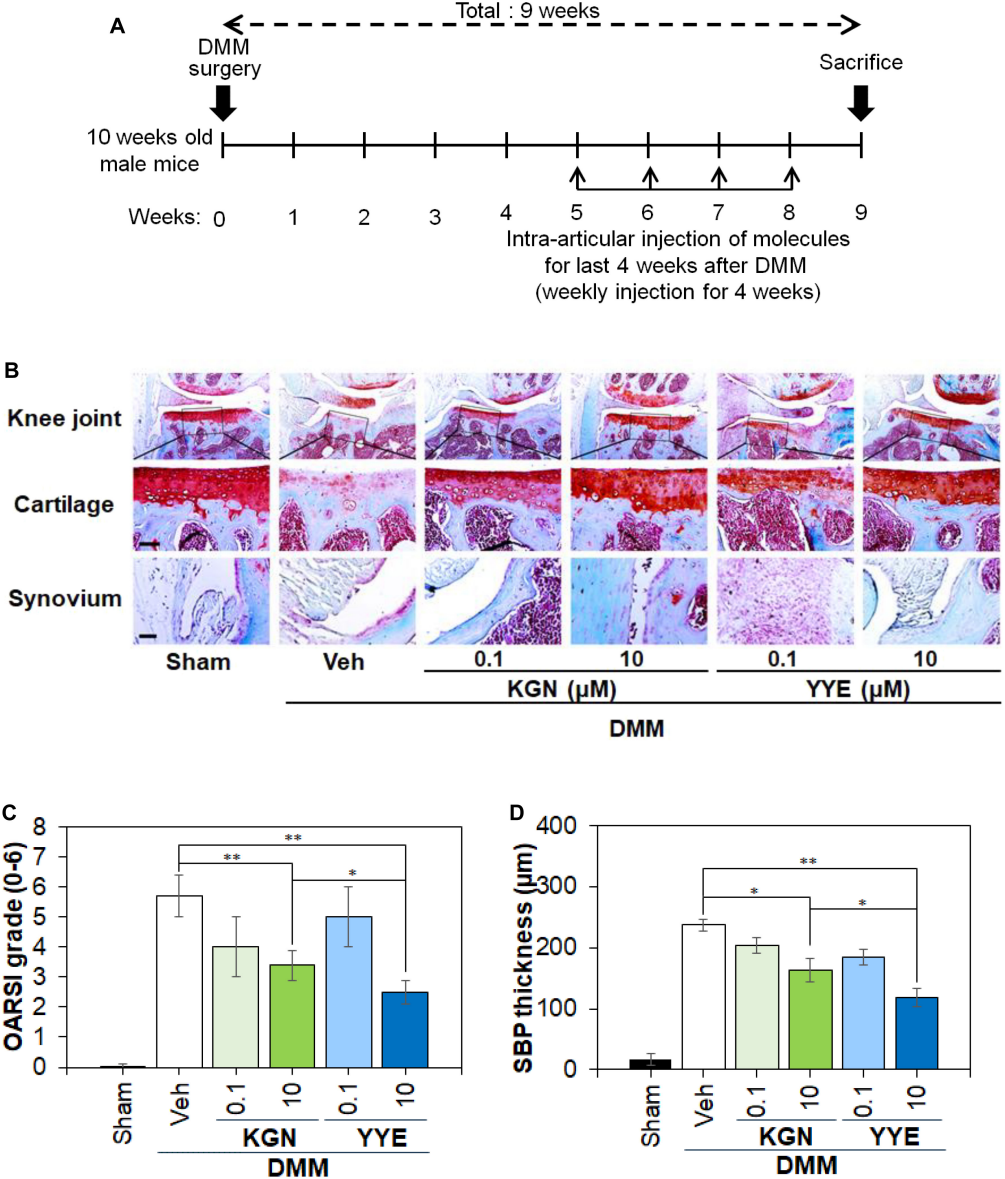
(A) Protocol for induction of an OA mouse model, followed by IA injections of chondrogenic promoter compound solutions (10 μl per mouse at 0.1 and 10 μM). *N* = 3 for each group. (B) Safranin O-stained images of the knee joint, cartilage, and synovium of mice. Scale bar, 25 μm. (C) OARSI grade. (D) SBP thickness. Sham and Veh (vehicle) indicate the cartilages of the healthy mice and OA mice with IA injection of PBS, respectively. KGN is used as a positive control compound. * and ** in (C) and (D) indicate *P* < 0.05 and *P* < 0.01, respectively, among the compared groups.

Stromal cell-derived factor-1 (sdf-1) is a chemokine to recruit MSCs and is oversecreted in OA patients in humans as well as mice [42,43]. Therefore, the IA injection of a drug that converts innate MSCs to chondrocytes might be a method for articular cartilage regeneration. Based on this scenario, the phase I clinical trial of KGN was carried out by IA injection of KGN [44]. In a similar strategy, IA injections of YYE into the mouse OA models were induced by DMM surgery. The administration of YYE was scheduled 5 weeks after the DMM surgery, entailing weekly IA injections of YYE during the latter half of the surgical period. In total, 4 IA injections of YYE were practiced to the mouse OA models, as illustrated in Fig. 7A. Notably, the application of YYE resulted in a dose-dependent improvement of articular cartilage degradation and other pathological indications within the affected knee joints (Fig. 7B to D and Fig. S3). In particular, OARSI grade was 5.7 for negative control (Veh; vehicle injection), whereas KGN and YYE injections (10 μM, 10 μl) reduced OARSI grade 3.4 and 2.6, respectively (Fig. 7C). OARSI grades are a measure of severity and extent of OA in the articular cartilage, while the total score ranges from 0 (Sham; intact cartilage) to 6 (very severe OA status) [31]. SBP thickness, which is also a measure of cartilage degeneration, was also reduced from 237 μm (negative control) to 163 μm and 119 μm by KGN and YYE injections, respectively (Fig. 7D) [45]. Consequently, the IA administration of YYE significantly improved the articular cartilage damage in the OA mouse model. When mice received YYE during the latter phase of the study, YYE seemed to foster cartilage regeneration better than KGN. To conclude, YYE can be an emerging compound exhibiting potent therapeutic efficacy subsequent to the onset of OA. In the context of in vivo cartilage regeneration, the differentiation of MSCs into chondrocytes must be complemented by the cells’ ability to migrate into and repopulate the damaged extracellular matrix. Recent studies have shown that chondrocyte motility is largely regulated by phosphatidylinositol 3-kinase/protein kinase B (PI3K/Akt) and extracellular signal-regulated kinase (ERK) signaling pathways, which promote actin cytoskeleton reorganization and enhance migratory activity [46]. This enables cells to invade sites of matrix degradation and participate in tissue repair. Such motile behavior is especially relevant in osteoarthritic cartilage, where inflammatory signals and altered matrix properties serve as chemoattractants that further stimulate chondrocyte migration [47]. Both articular and nasal chondrocytes have demonstrated these migratory capabilities, supporting their integration into host tissue and contributing to the structural restoration of cartilage [48]. To summarize, the regenerative potential of MSC-derived chondrocytes depends not only on lineage commitment but also on their intrinsic migratory responses, which are critical mechanisms for effective cartilage repair.

## Conclusion

In this study, YYE was developed as a potent chondrogenic promoter. YYE exhibited no toxicity at its effective concentration of 10 μM. Chondrogenic biomarkers at both the mRNA level (COL II, COMP, and ACAN) and protein level (COL II), as well as Alican Blue (sGAG) and Safrain O (proteoglycan) staining, indicated that YYE is comparable to KGN, the postive control molecule. Notably, YYE induced significantly higher COMP mRNA expression than KGN (*P* < 0.05). Both in silico modeling and enhanced expression of CBFβ and RUNX1 suggested that YYE and KGN share a similar mechanism in promoting MSC chondrogenesis. Although KGN has lower synthetic cost and a simpler manufacturing process—being synthesized via the reaction between phthalic anhydride and 4-aminobiphenyl—YYE may offer advantages in clinical translatability. YYE is a simple tripeptide expected to degrade into amino acids, thereby reducing toxicity, whereas the degradation products of KGN, 4-aminobiphenyl and phthalic acid, are associated with carcinogenicity and developmental and reproductive toxicity, respectively [49,50]. In vivo application in an OA mouse model demonstrated excellent cartilage regeneration for both compounds, as evidenced by improved OARSI grades and SBP thickness, with YYE performing significantly better than KGN (*P* < 0.05). The study proposes that YYE can be a promising drug candidate for OA treatments.

## Ethical Approval

All procedures involving animals were conducted in adherence to the highest ethical standards. The Institutional Animal Care and Use Committees (IACUC) of Ewha Womans University provided approval (protocol nos.: IACUC 21-076 and 24-081) for all animal-related protocols. These experimental procedures were executed in accordance with the guidelines laid out by the National Research Council.

## Data Availability

Data that support the findings of this study can be provided by the corresponding authors.
