# Supplementary material for: Tyr-Tyr-Glu Tripeptide for Regeneration of Articular Cartilage through Chondrogenic Differentiation of Mesenchymal Stem Cells
Source: Biomater Res. 2025 Nov 14;29:0272. doi: 10.34133/bmr.0272 (PMC12615153; doi:10.34133/bmr.0272)

Supporting Information

**Tyr-Tyr-Glu tripeptide for regeneration of articular cartilage through chondrogenic differentiation of mesenchymal stem cells**

Ye Lin Kim,^1^ Soyoun Um,^1^ Madhumita Patel,^1^ Yeon-Ju Jung,^1^ Sun-Shin Cha,^1,2^ Jihee Kim,^3^ Soo Young Lee,*^3^ and Byeongmoon Jeong *^1^

1 Department of Chemistry and Nanoscience, Gradutate Program in Innovative Biomaterials Convergence, Ewha Womans University, 52 Ewhayeodae-gil, Seodaemun-gu, Seoul 03760, Korea

2 R&D Division, TODD Phaarm Co. Ltd, Seoul 03760, Korea

3 Department of Life Sciences and Multitasking Macrophage Research Center, Ewha Womans University, 52 Ewhayeodae-gil, Seodaemun-gu, Seoul 03760, Korea

*Address correspondence to

[bjeong@ewha.ac.kr](mailto:bjeong@ewha.ac.kr) & leesy@ewha.ac.kr

**Table S1.** Primer sequences and PCR conditions for real time RT-PCR.

| Genes | Primer sequences | Annealing  Temp. (℃) |
| --- | --- | --- |
| COL II | F: 5’-CACTGGCAGTGGCGAGGTCAG-3’  R: 5’-CACTGGCAGTGGCGAGGTCAG-3’ | 65.0  62.8 |
| SOX 9 | F: 5’-GCAGCGAAATCAACGAGAAAC-3’  R: 5’-TCCAAACAGGCAGAGAGATTTAG-3’ | 55.0  54.5 |
| COMP | F: 5’-AGCAGATGGAGCAAACGTATTG-3’  R: 5’-ACAGCCTTGAGTTGGATGCC-3’ | 55.6  58.0 |
| ACAN | F: 5’-CAGAGCAAGACTCTGTCTCAAA-3’  R: 5’-GGGACAAGAGCCTCATCAAA-3’ | 54.5  54.8 |
| COL X | F: 5’-CAAGGCACCATCTCCAGGAA-3’  R: 5’-AAAGGGTATTTGTGGCAGCATATT-3’ | 57.2  55.3 |
| GAPDH | F: 5’- CTCCTCACAGTTGCCATGTA-3’  R: 5’- GTTGAG5CACAGGGTACTTTATTG-3’ | 54.5  53.9 |

* COL II, SOX 9, COMP, ACAN, COL X, and GAPDH indicate collagen type II, SRY-box 9, cartilage oligomeric matrix protein, aggrecan, collagen type X, and glyceraldehyde 3-phosphate dehydrogenase, respectively. F and R indicate forward and reverse primers, respectively.

**Fig. S1.** HPLC traces of tripeptides.

| **HPLC trace** |
| --- |
| **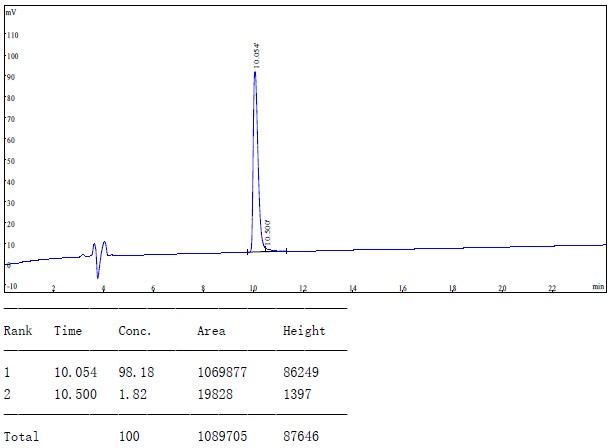AAE Purity 98.2%** |
| **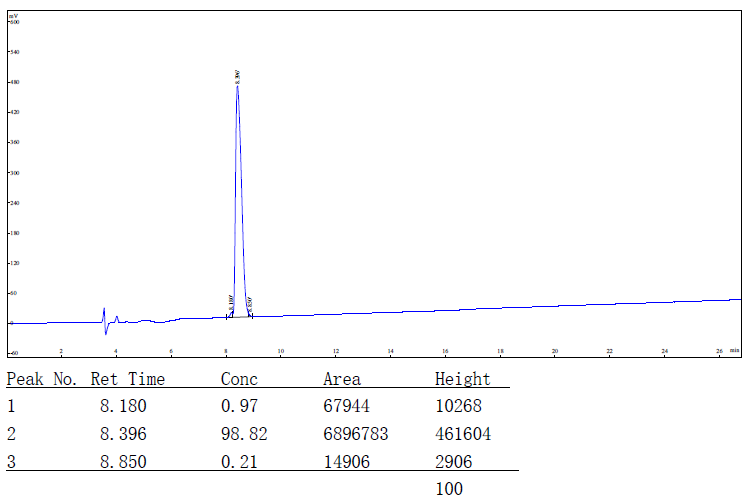FFE Purity 98.8%** |

| **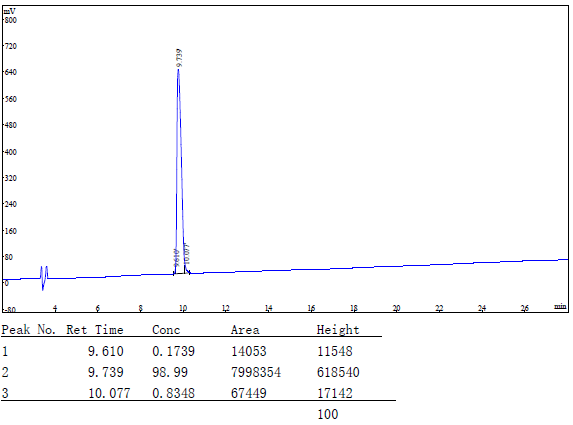IFE Purity 99.0%** |
| --- |
| **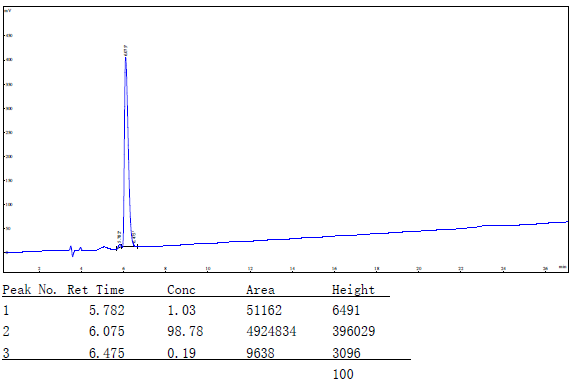LFE Purity 98.8%** |

| 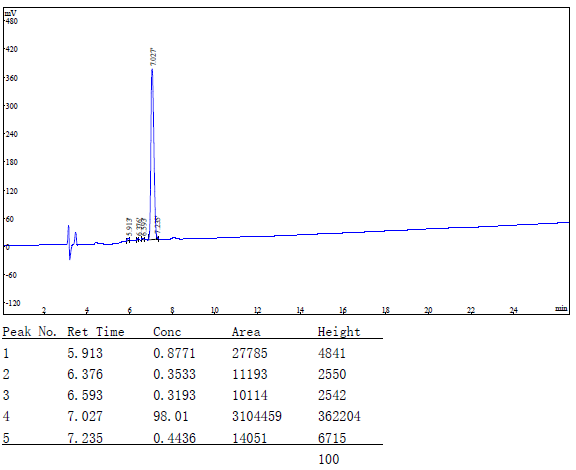**MFE Purity 98.0%** |
| --- |
| 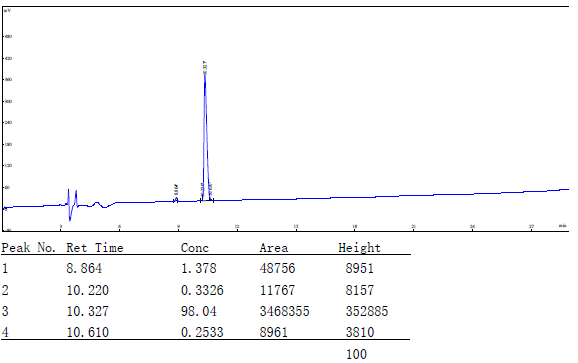**VFE Purity 98.0%** |

| 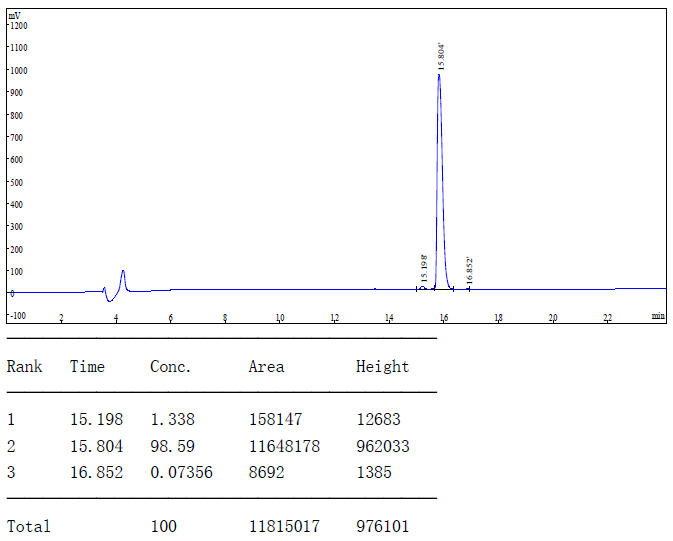**WFE Purity 98.6%** |
| --- |
| 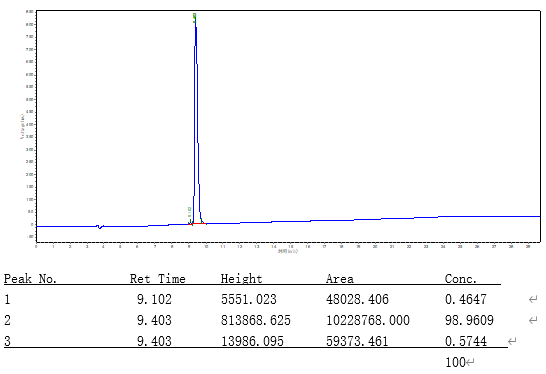**YFE Purity 98.9%** |

| 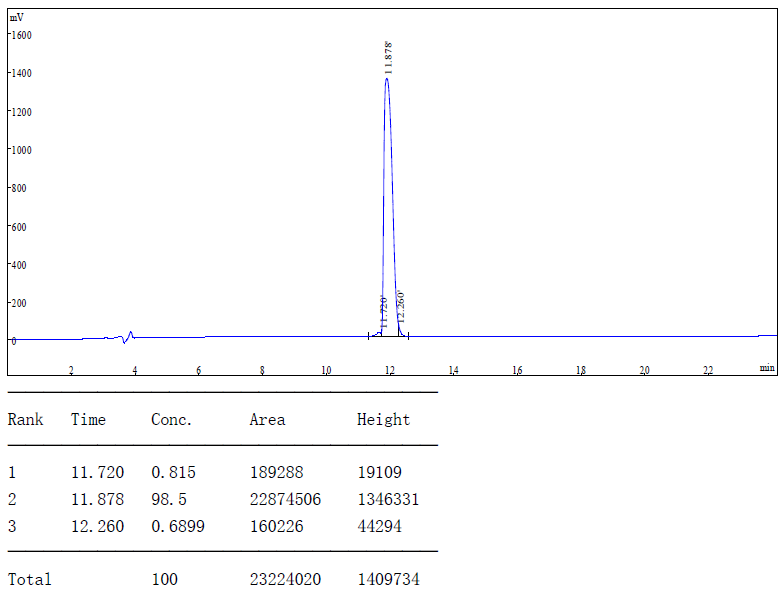**FRD Purity 98.5%** |
| --- |
| 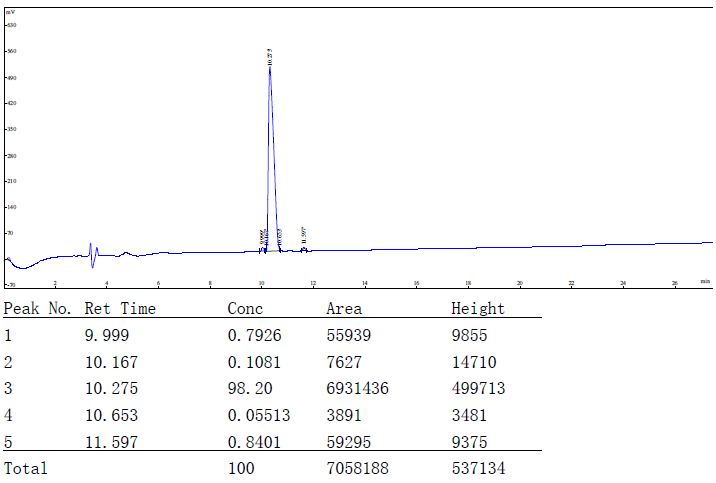**FLE Purity 98.2%** |
| 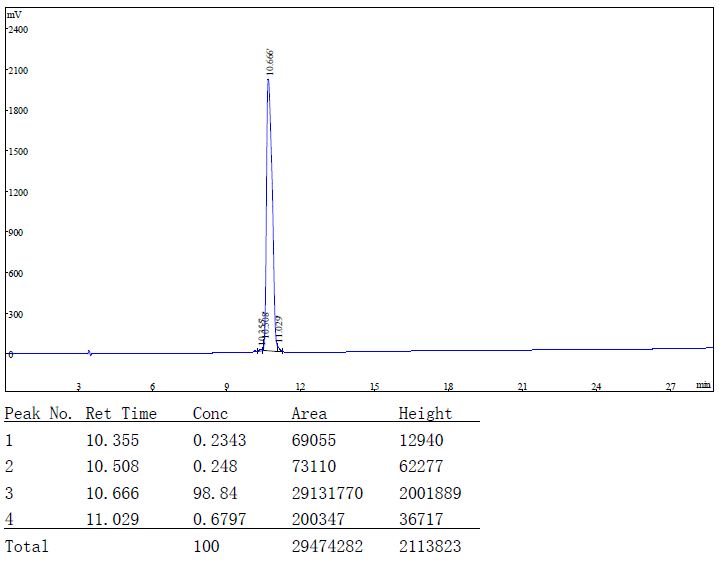**WLE Purity 98.8%** |
| 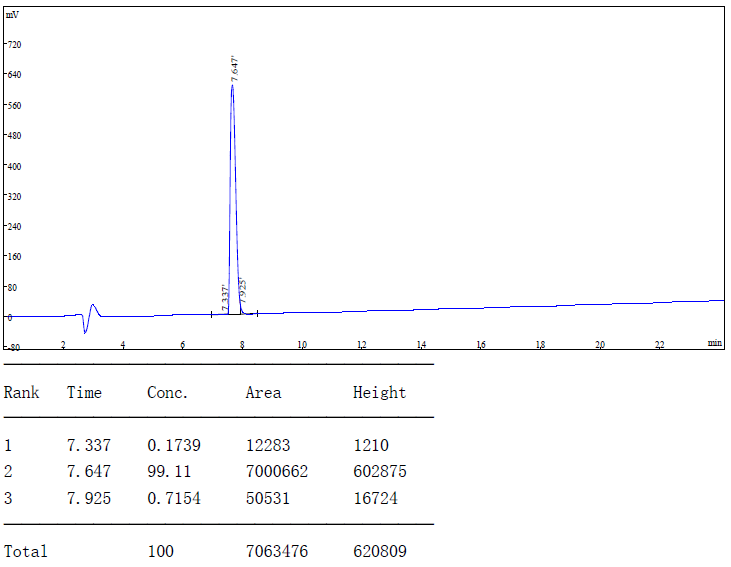**YID Purity 99.1%** |
| 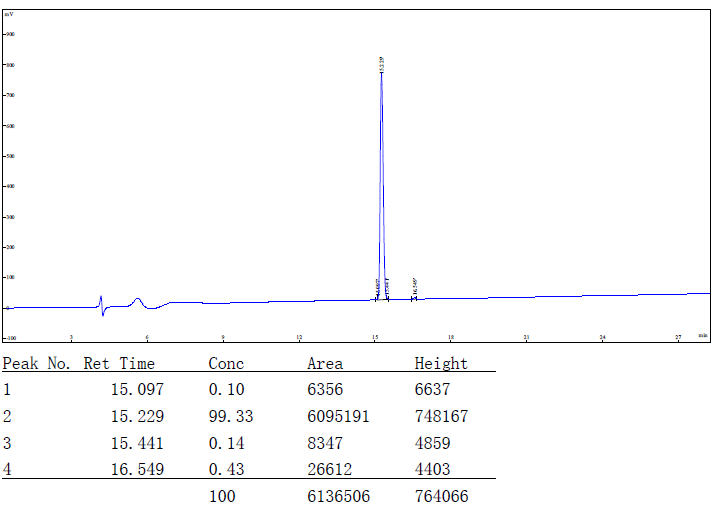**YYE Purity 99.3%** |

**Fig. S2.** Cell viability of TMSCs after three days of incubation assayed by the Live/Dead kit.


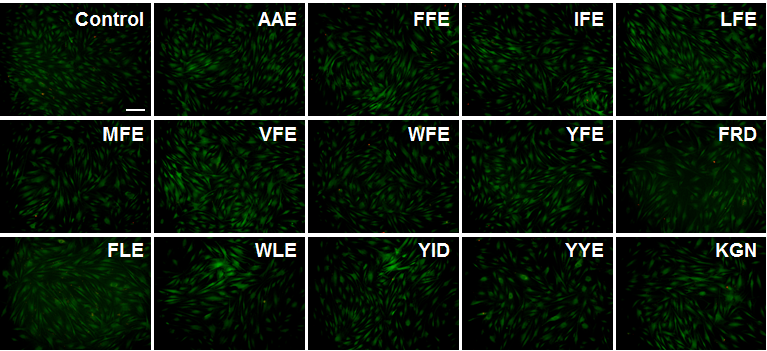


**Fig. S3.** In vivo assay for articular cartilage regeneration. a) osteophyte maturity. b) synovitis assay.


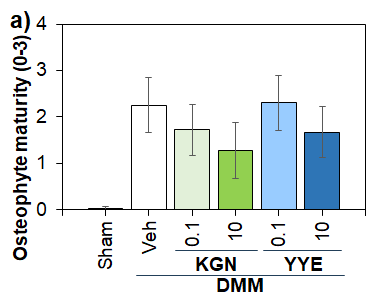

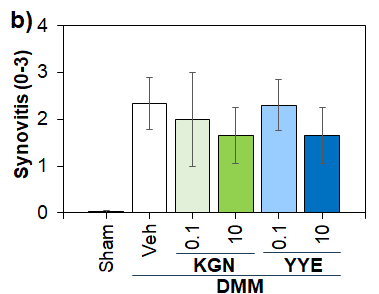

Supplement: Supplementary 1 — Figs. S1 to S3 Table S1 [file bmr.0272.f1.docx]
